# Effect of workplace violence on health workers injuries and workplace absenteeism in Bangladesh

**DOI:** 10.1186/s41256-023-00316-z

**Published:** 2023-08-22

**Authors:** Md. Shahjalal, Md. Parvez Mosharaf, Rashidul Alam Mahumud

**Affiliations:** 1 Global Health Institute, North South University, Dhaka, Bangladesh; 2 Department of Public Health, North South University, Dhaka, Bangladesh; 3 Research Rats, Dhaka, Bangladesh; 4 School of Business and Centre for Health Research, University of Southern Queensland, Toowoomba, QLD Australia; 5 Health Economics and Health Technology Assessment Unit, NHMRC Clinical Trials Centre, Faculty of Medicine and Health, The University of Sydney, Camperdown, NSW Australia

**Keywords:** Workplace violence, Health workers, Injury, Absenteeism, Bangladesh

## Abstract

**Background:**

Workplace violence (WPV) is an emerging problem for health workers (HWs) and a global concern in health systems. Scientific literatures infer that WPV against HWs is often attributed to workplace injuries and absenteeism, leading to a series of adverse consequences. Therefore, this study aimed to investigate the prevalence of workplace injuries and absenteeism due to WPV among Bangladeshi HWs and its association with factors related to health facilities, work environments, and rotating shift work.

**Methods:**

This study used participants who had experienced WPV, including medical doctors, nurses, or any form of medical staff. A total of 468 victim HWs were added in the analytical exploration. Participants were generated from our previous cross-sectional study of 1081 Bangladeshi HWs. A logistic regression model was used to find the association between workplace injuries and absenteeism due to WPV among HWs and associated factors.

**Results:**

The prevalence of workplace injuries and absenteeism due to WPV among HWs were 14.10% (95% CI 11.23–7.57) and 22.44% (95% CI 18.87–26.45), respectively. Injury incidence was higher among males (17.67%) and young HWs (20.83%). Workplace absenteeism was more common among male HWs (25%) and those working in public hospitals (23.46%). The magnitude of injuries and absenteeism varied significantly by hospital departments. Workplace injury was significantly higher among HWs who worked in the emergency (AOR = 21.53, 95% CI 2.55–181.71), intensive care (AOR = 22.94, 95% CI 2.24–234.88), surgery (AOR = 17.22, 95% CI 1.96–151.39), and gynecology & obstetrics departments (AOR = 22.42, 95% CI 2.25–223.07) compared with other departments. The burden of work-related absenteeism was significantly associated with HWs who worked in the emergency (AOR = 4.44, 95% CI 1.56–12.61), surgery (AOR = 4.11, 95% CI 1.42–11.90), and gynecology and obstetrics departments (AOR = 5.08, 95% CI 1.47–17.50).

**Conclusions:**

This study observed a high prevalence of workplace injuries and absenteeism among HWs due to WPV across hospital departments, including emergency, surgery, intensive care and gynecology & obstetrics units. Policymakers should incorporate suitable strategies into Bangladesh’s national health policy to combat violence in healthcare settings.

**Supplementary Information:**

The online version contains supplementary material available at 10.1186/s41256-023-00316-z.

## Introduction

Workplace violence (WPV) against health workers (HWs) is described as aggression when staff members are abused, threatened, or attacked in circumstances related to their work [1]. It has gradually become a common global occupational hazard that negatively affects health care systems [2]. According to the World Health Organization (WHO), WPV is particularly prevalent in health care systems compared with other work settings worldwide [3]. The health sector encompasses various jobs, ranging from home care aid to hospital services; therefore, it poses risks of violence due to interaction between HWs and patients [2, 3]. A shocking finding revealed by international studies is that the health system has the highest risk of WPV [1–4] and in some cases, causes physical injuries and even death among HWs [4–7]. Even more concerning is that the severity of these injuries and fatalities is prevalent in both developed and developing countries [7–10].

According to the U.S. Bureau of Labor Statistics, 73% of violence-related injuries occurred among HWs in 2018 [8]. A recent systematic review concluded that approximately 70% of Chinese HWs were injured and 12.8% of them died between 2004 and 2018 [9]. A similar experience was observed in Indian hospitals, whereas about 18% of pre-hospital providers such as emergency medical technicians, paramedics, emergency doctors and nurses among victims were injured due to WPV [10]. Considering this burden, Bangladesh also has similar experiences concerning its HWs [7]. Several studies reported that the incidence of workplace injuries varies among hospital departments [11–16]. In specific, the incidents of injuries are substantially higher in emergency [11–13], surgery [14], intensive care [15], and psychiatric departments [5, 16] compared with other hospital departments.

A growing body of evidence showed that workplace injuries negatively impact HWs personally and professionally and has a long-term implication for HWs and patients [6, 16–19]. The previous studies presented that these adverse effects influence job dissatisfaction [17, 19, 20], low employee engagement [6, 19], impaired work functioning [17, 19], leading to insecurity [5, 21], more sickness absence [18, 22, 23], and fear of returning to work [2, 6, 19]. For instance, a Finnish study reported that HWs who were bullied in their workplace were one and a half times more likely to take sick leave than their non-bullied peers [23]. A person's work not only relates to a fundamental duty and obligation to provide for themselves but is also believed to play an essential role in defining their identity, self-esteem, and self-image [24, 25]. In the same way, the work environment is essential for employees' potential performance where they spend most of their time. For example, HWs devote a substantial part of their days in the hospital to delivering healthcare services, and their workability and productivity correlate with a safe and sound workplace [17]. The impact of WPV and its adverse outcomes lead to a huge health system burden, including workforce shortage, quality of care services [6, 7], particularly in developing countries[4, 10, 21, 26]. In this regard, Bangladesh is not an exception [7, 27].

Human resources for health are one of the major setbacks to implementing the Sustainable Development Goals (SDGs) in Bangladesh [28]. The latest Bangladesh Ministry of Health and Family Welfare report showed that there are only 6 doctors, nurses, and midwives per 10,000 people in the country which is the second lowest in South Asia [29]. With other underlying problems, such as patient overload, a lack of healthcare resources, a lack of interpersonal communication, insufficient security measures, and the shortage of the country’s HWs hinders meeting patients’ demands for quality healthcare services [28]. In the low- and middle-income countries setting, including Bangladesh, there is an urgent need to find adequate solutions to this issue since workplace injuries in health facilities are likely to worsen the impending work absenteeism, and the shortage of HWs will exacerbate the deterioration in the quality of care.

It has been documented extensively elsewhere that workplace injuries due to WPV are prevalent in various hospital departments such as emergency and surgery unit and has an impact on higher work absenteeism [6, 17, 19]. However, it is unclear what the consequences of WPV are on injuries and absenteeism among Bangladeshi HWs. This present study was nested from a previous study that investigated the prevalence and associated factors of WPV among Bangladeshi HWs [27]. The parent study did not assess the impact of WPV on workplace injuries and absenteeism among HWs in hospital departments.

To date, to our knowledge, no research has examined the consequences of WPV on injuries and absenteeism by working hospital departments among Bangladeshi HWs. This knowledge gap must be addressed by policymakers and other stakeholders to develop evidence-based measures to tackle workplace injuries and absenteeism among HWs to ensure safe workplaces and adequate healthcare delivery to meet SDG target 3.8 which emphasises achieving Universal Health Coverage (UHC) by 2030*.* In addition, SDG indicator 3.8.1 promotes access to quality essential healthcare services for all and indicator 3.C.1 focuses on retaining health workforces in developing countries like Bangladesh [30]. For this reason, it is essential for policymakers to understand the impact of WPV on workplace injuries and absenteeism among Bangladeshi HWs. This study, therefore, aims to investigate the prevalence of workplace injuries and absenteeism due to WPV and its association across working departments among Bangladeshi HWs.

## Methods

### Study design and participants

**Fig. 1:**
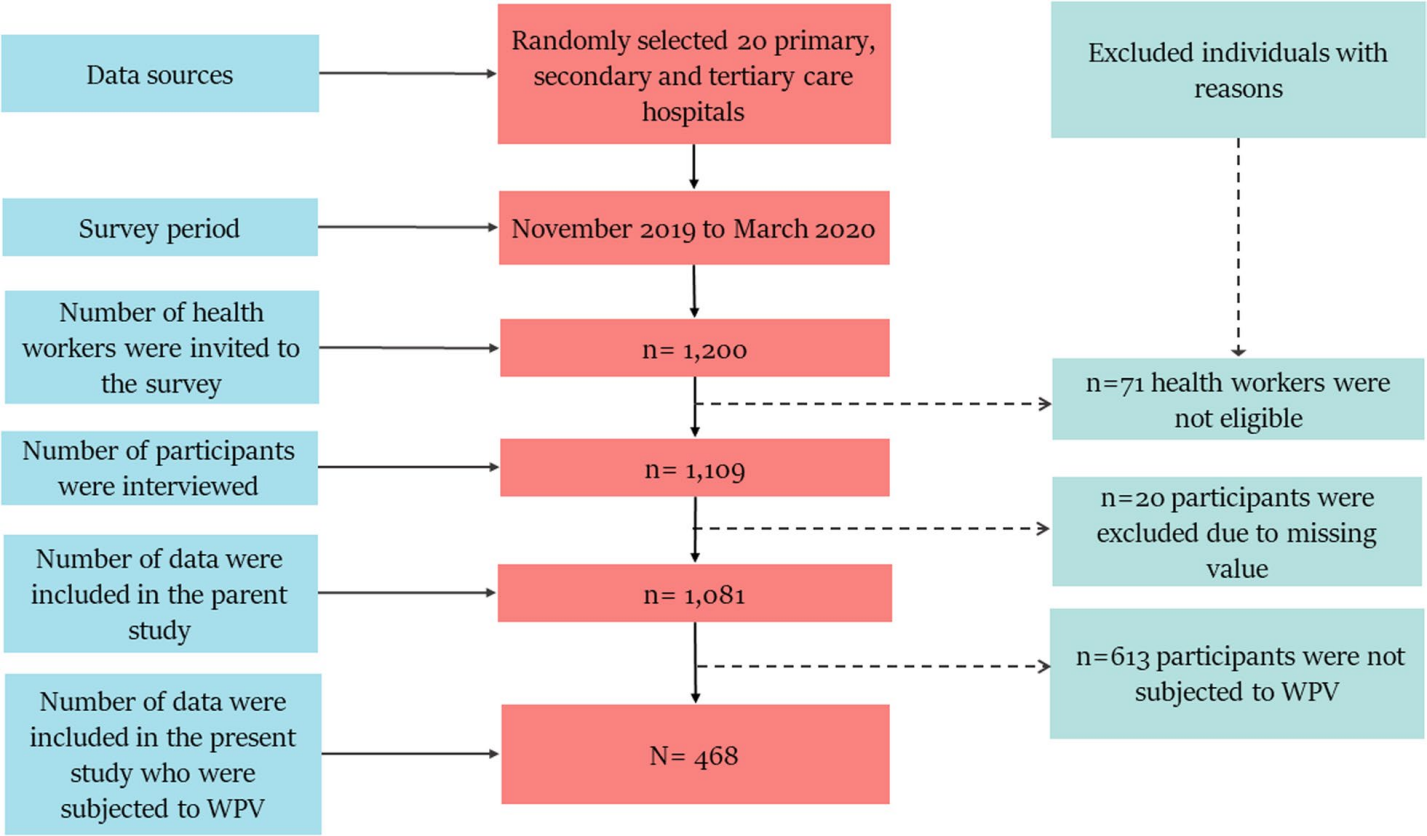
Distribution of study participants

This study is a part of an initial cross-sectional assessment of WPV among HWs in Bangladesh [27]. The survey was conducted among HWs from randomly selected twenty public and private hospitals across eight administrative divisions between November 2019 and March 2020. The study subject included registered physicians, nurses, paramedical staff, medical technicians and attendants working in the hospital with at least one year of experience. A self-administered questionnaire based on the “Workplace Violence Survey Questionnaire (2003, ILO/ICN/WHO/PSI)” was used to assess experiences of WPV and its consequences for work functioning over the past 12 months [31]. Before participants filled out the questionnaires, data collectors informed them of the purpose of the study and that it was confidential and voluntary. However, the present study comprised a sample of 468 HWs who were subjected to WPV among 1081 participants in the 12 months prior to the survey [27]. The process of data selection, identification and inclusion flow diagram is presented in Fig. 1.

### Measures

#### Outcome measures

Workplace injuries and absenteeism due to WPV were considered the outcome variables and measured based on self-reported responses. Workplace injuries (for example, slight bodily injury, soft tissue contusion, nose bleeding) due to WPV was assessed with a response option of ‘yes’ vs ‘no’: ‘Were you injured as a result of WPV?’. Workplace absenteeism due to WPV (such as take off from work for days/weeks/months) was measured based on the question ‘Did you take off from your work after being a workplace victim in the last 12 months?’. This response was dichotomised (1 = ‘yes’ if the participants reported workplace absenteeism due to WPV or 0 = ‘no’ otherwise).

#### Explanatory variables

Following previous literature, several sociodemographic and occupational variables were considered in the analytical exploration (Table 1). A detailed definition of the explanatory variables is presented in Additional file 1.

**Table 1:** Participant’s sociodemographic characteristics and their workplace injuries and absenteeism due to WPV (n = 468)

Participants’ characteristics	Number of participants	Injured due to WPV	Work absenteeism due to WPV
	n (%)	% (95% CI)	% (95% CI)
*Gender*			
Male	232 (49.57)	17.67 (13.27–23.14)	25.00 (19.84–30.99)
Female	236 (50.43)	10.59 (7.25–15.22)	19.92 (15.29–25.52)
*p* Value		0.028	0.187
*Age in years*			
20–25	48 (10.26)	20.83 (11.57–34.6)	16.67 (8.55–29.98)
26–30	119 (25.43)	14.29 (9.06–21.81)	22.69 (16.03–31.09)
31–35	138 (29.49)	13.77 (8.95–20.60)	21.01 (15.00–28.63)
36–40	101 (21.58)	10.89 (6.13–18.63)	19.80 (13.13–28.74)
> 40	62 (13.25)	14.52 (7.72–25.64)	33.87 (23.21–46.46)
*p* Value	0.613		0.189
*Health worker*			
Doctor	356 (76.07)	11.24 (8.34–14.97)	23.31 (19.20–28.00)
Nurse	103 (22.01)	21.36 (14.48–30.34)	18.45 (12.07–27.15)
Other HWs	9 (1.92)	44.44 (17.63–74.93)	33.33 (11.08–66.74)
*p* Value		0.001	0.424
*Health facility level*			
Primary	55 (11.75)	16.36 (8.73–28.59)	21.82 (12.81–34.64)
Secondary	59 (12.61)	23.73 (14.57–36.21)	23.73 (14.57–36.21)
Tertiary	354 (75.64)	12.15 (9.13–15.99)	22.32 (18.27–26.96)
*p* Value	0.053		0.965
*Health facility type*			
Private	144 (30.77)	16.67 (11.42–23.68)	20.14 (14.36–27.50)
Public	324 (69.23)	12.96 (9.72–17.09)	23.46 (19.15–28.39)
*p* Value		0.288	0.427
*Experience in years*			
< 4	134 (28.63)	15.67 (10.43–22.87)	17.91 (12.29–25.36)
4–6	113 (24.15)	12.39 (7.47–19.86)	24.78 (17.67–33.58)
7–10	83 (17.74)	19.28 (12.14–29.21)	21.69 (14.10–31.85)
> 10	138 (29.49)	10.87 (6.65–17.27)	25.36 (18.79–33.29)
*p* Value		0.312	0.451
*Department*			
General medicine	23 (4.91)	4.35 (0.61–25.32)	13.04 (4.25–33.62)
Surgery	114 (24.36)	13.16 (8.08–20.71)	26.32 (19.04–35.17)
Emergency	123 (26.28)	20.33 (14.11–28.38)	33.33 (25.56–42.13)
Intensive care	26 (5.56)	19.23 (8.22–38.76)	11.54 (3.76–30.35)
Pediatrics	42 (8.97)	7.14 (2.32–19.98)	16.67 (8.14–31.09)
Gynecology & obstetrics	37 (7.91)	18.92 (9.28–34.74)	27.03 (15.18–43.39)
Orthopedics	21 (4.49)	9.52 (2.38–31.21)	9.52 (2.38–31.21)
Management	19 (4.06)	15.79 (5.16–39.23)	21.05 (8.11–44.62)
Other departments	63 (13.46)	7.94 (3.33–17.72)	7.94 (3.33–17.72)
*p* Value		0.193	0.003
*Rotating shift work*			
No	179 (38.25)	11.73 (7.77–17.34)	12.85 (8.68–18.61)
Yes	289 (61.75)	15.57 (11.82–20.23)	28.37 (23.46–33.86)
*p* Value		0.246	< 0.001
*Workplace location*			
Dhaka division	91 (19.44)	12.09 (6.81–20.55)	20.88 (13.72–30.46)
Chittagong division	59 (12.61)	5.08 (1.64–14.65)	15.25 (8.12–26.83)
Sylhet division	45 (9.62)	11.11 (4.69–24.10)	26.67 (15.79–41.36)
Khulna division	54 (11.54)	16.67 (8.89–29.07)	18.52 (10.25–31.15)
Rangpur division	41 (8.76)	17.07 (8.35–31.76)	31.71 (19.36–47.31)
Barisal division	57 (12.18)	19.30 (11.00–31.62)	28.07 (17.94–41.05)
Rajshahi division	59 (12.61)	13.56 (6.92–24.88)	23.73 (14.57–36.21)
Mymensingh division	62 (13.25)	19.35 (11.32–31.10)	19.35 (11.32–31.10)
*p* Value		0.343	0.508
Overall	468 (100)	14.10 (11.23–17.57)	22.44 (18.87–26.45)

*p* value = the probability value. The *p* value was generated using chi-square test

### Statistical analysis

In the descriptive analyses, the characteristics of the study participants were expressed using frequencies (n) and percentages (%). In addition, the dependent variables (workplace injuries and absenteeism due to WPV) were characterised as binary responses (yes vs. no). The chi-square test was used to perform bivariate association between the sociodemographic factors and outcome measures. The statistical significance was considered by a *p* value ≤ 0.05 and corresponding precision estimate with a 95% confidence interval (CI). In the analytical exploration, adjusted logistic regression models were employed to examine the association of having an incident of injury and work absenteeism due to WPV on participant’s socio-demographic and work-related factors among HWs in Bangladesh. The potential explanatory variables were added in the final model (adjusted) only if any label of the covariate was statistically significant with a *p* value of 25% or less in the unadjusted model. All statistical analyses were performed using the statistical software Stata/SE 16 (StataCorp, College Station, Texas, USA).

## Results

### Participant’s background characteristics

Table 1 presents a summary of participant characteristics. A total of 468 participants were included in this study. The average age of the participants was 30.83 years (Standard Deviation, SD = 6.75 years). Half of the participants were female (n = 236, 50.43%). Two-thirds of participants were medical doctors (n = 356, 76.07%) and worked in tertiary hospitals (n = 354, 75.64%). Approximately 70% of participants were government sector employees (n = 324, 69.23%). About thirty percent of participants had more than 10 years of work experience in clinical practice (n = 138, 29.49%). One-fourth of participants worked in the emergency (n = 123, 26.28%) and surgery departments (n = 114, 24.36%). Approximately 62% of participants worked on a rotating shift schedule (n = 289, 61.75%).

### Characteristics of workplace injury and absenteeism due to WPV

The prevalence of injury due to WPV among HWs was 14.10% (n = 66) (95% CI 11.23–17.57). Of them, injuries were more prevalent among males (n = 41, 17.67%), secondary-level hospital employees (n = 14, 23.73%), and private hospitals (n = 24, 16.67%). It was also higher among HWs with 7–10 years of working experience in hospital (n = 16, 19.28%), emergency department staff (n = 25, 20.33%), worked in rotating shift (n = 45, 15.57%), and were from the Mymensingh division (n = 66, 19.35%) (Table 1).

As a result of being injured, 22.44% (n = 105) (95% CI 18.87–26.45) of HWs were absent from their regular work duties. The majority of absent HWs were males (n = 58, 25%) and over the age of 40 years (n = 21, 33.87%). In addition, the HWs who worked in public (n = 76, 23.46%) and secondary-level hospitals (n = 14, 23.73%) were frequently absent in their workplace. However, we also observed that working department (*p* = 0.003) and shift work (*p* < 0.001) were statistically significant (Table 1).

### Factors associated with workplace injury and absenteeism

Table 2 shows the logistic regression analysis on having an injury and work absenteeism due to WPV among HWs. The magnitude of having injury due to WPV varied significantly by HW’s working departments. For example, having an injury, WPV was significantly associated with HWs who worked in the emergency (AOR = 21.53, 95% CI 2.55–181.71; *p* = 0.005); surgery (AOR = 17.22, 95% CI 1.96–151.39; *p* = 0.010); gynecology & obstetrics departments (AOR = 22.42, 95% CI 2.25–223.07; *p* = 0.008), and intensive care unit (AOR = 22.94, 95% CI 2.24–234.88; *p* = 0.008) compared to HWs who worked at other departments.

**Table 2:** The logistic regression analysis of injury and work absenteeism due to WPV among HWs

Participants’ characteristics	Injured due to WPV				Work absenteeism due to WPV			
	Unadjusted model		Adjusted model		Unadjusted model		Adjusted model	
	OR (95% CI)	*p* Value	OR (95% CI)	*p* Value	OR (95% CI)	*p* Value	OR (95% CI)	*p* Value
*Female (ref* = *male)*	0.55 (0.32–0.94)	0.029	0.50 (0.27–0.93)	0.029	0.75 (0.48–1.15)	0.188	0.81 (0.5–1.31)	0.397
*Age group in years (ref* = *20–25)*								
26–30	0.63 (0.27–1.50)	0.301	0.29 (0.03–3.35)	0.323	1.47 (0.61–3.51)	0.389	1.25 (0.19–8.10)	0.817
31–35	0.61 (0.26–1.42)	0.248	0.40 (0.06–2.81)	0.359	1.33 (0.56–3.15)	0.517	0.84 (0.17–4.19)	0.834
36–40	0.46 (0.18–1.18)	0.109	0.56 (0.11–2.83)	0.484	1.23 (0.50–3.05)	0.647	0.42 (0.11–1.65)	0.214
> 40	0.65 (0.24–1.74)	0.387	0.59 (0.18–1.91)	0.375	2.56 (1.02–6.45)	0.046	0.44 (0.19–1.02)	0.057
*Health worker (ref* = *other health workers)*								
Doctor	0.16 (0.04–0.61)	0.008	0.005 (0.003–0.07)	< 0.001	0.61 (0.15–2.48)	0.488	–	–
Nurse	0.34 (0.08–1.37)	0.130	0.035 (0.003–0.474)	0.012	0.45 (0.10–1.97)	0.291	–	–
*Health facility level (ref* = *tertiary)*								
Primary	1.42 (0.65–3.09)	0.384	1.30 (0.48–3.51)	0.603	0.97 (0.49–1.93)	0.934	–	–
Secondary	2.25 (1.14–4.44)	0.019	1.76 (0.76–4.05)	0.185	1.08 (0.57–2.07)	0.810	–	–
*Public health facility (ref* = *private)*	0.74 (0.43–1.28)	0.289	0.94 (0.47–1.89)	0.863	1.22 (0.75–1.97)	0.428	–	–
*Experience in years (ref* = > *10 years)*								
< 4	1.52 (0.75–3.10)	0.245	1.81 (0.3–10.99)	0.520	0.64 (0.36–1.15)	0.138	0.50 (0.10–2.38)	0.382
4–6	1.16 (0.53–2.52)	0.708	1.21 (0.25–5.84)	0.811	0.97 (0.55–1.72)	0.916	1.33 (0.35–5.10)	0.681
7–10	1.96 (0.91–4.21)	0.085	2.29 (0.64–8.20)	0.201	0.81 (0.43–1.56)	0.536	1.23 (0.41–3.62)	0.712
*Department (ref* = *other departments)*								
General medicine	0.53 (0.06–4.77)	0.569	4.09 (0.22–76.20)	0.345	1.74 (0.38–7.95)	0.475	2.51 (0.51–12.29)	0.257
Surgery	1.76 (0.61–5.09)	0.298	17.22 (1.96–151.39)	0.010	4.14 (1.52–11.31)	0.006	4.11 (1.42–11.90)	0.009
Emergency	2.96 (1.07–8.15)	0.036	21.53 (2.55–181.71)	0.005	5.80 (2.16–15.57)	< 0.001	4.44 (1.56–12.61)	0.005
Intensive care	2.76 (0.73–10.51)	0.136	22.94 (2.24–234.88)	0.008	1.51 (0.33–6.85)	0.591	1.24 (0.26–5.93)	0.784
Pediatrics	0.89 (0.20–3.95)	0.881	9.52 (0.81–112.13)	0.073	2.32 (0.68–7.87)	0.177	3.08 (0.84–11.31)	0.090
Gynecology & Obstetrics	2.71 (0.79–9.25)	0.112	22.42 (2.25–223.07)	0.008	4.30 (1.34–13.80)	0.014	5.08 (1.47–17.50)	0.010
Orthopedics	1.22 (0.22–6.82)	0.820	10.56 (0.78–143.09)	0.076	1.22 (0.22–6.82)	0.820	1.00 (0.17–5.92)	0.998
Management	2.18 (0.47–10.09)	0.321	25.15 (2.02–312.52)	0.012	3.09 (0.74–12.95)	0.122	3.20 (0.69–14.93)	0.138
*Rotating shift work (ref* = *no)*	1.39 (0.80–2.42)	0.248	2.76 (1.16–6.55)	0.021	2.69 (1.62–4.46)	< 0.001	2.61 (1.43–4.75)	0.002
*Workplace location* *(ref* = *Mymensingh division)*								
Dhaka division	0.57 (0.23–1.40)	0.221	0.58 (0.21–1.57)	0.280	1.10 (0.49–2.47)	0.818	1.16 (0.49–2.74)	0.737
Chittagong division	0.22 (0.06–0.84)	0.026	0.16 (0.04–0.71)	0.015	0.75 (0.29–1.94)	0.552	0.77 (0.28–2.11)	0.610
Sylhet division	0.52 (0.17–1.60)	0.255	0.59 (0.17–2.03)	0.401	1.52 (0.61–3.78)	0.372	1.94 (0.73–5.17)	0.187
Khulna division	0.83 (0.32–2.16)	0.708	0.79 (0.26–2.37)	0.671	0.95 (0.37–2.40)	0.909	1.01 (0.37–2.76)	0.979
Rangpur division	0.86 (0.31–2.40)	0.770	0.93 (0.30–2.93)	0.906	1.93 (0.78–4.81)	0.156	1.96 (0.74–5.23)	0.178
Barisal division	1.00 (0.40–2.48)	0.994	0.89 (0.31–2.49)	0.818	1.63 (0.69–3.82)	0.265	1.72 (0.69–4.31)	0.247
Rajshahi division	0.65 (0.25–1.73)	0.393	0.56 (0.19–1.68)	0.302	1.30 (0.54–3.09)	0.559	1.54 (0.60–3.94)	0.366

The potential explanatory variables were added in the adjusted model only if any label of the covariate was statistically significant with a *p* value at 25% or less in the unadjusted model

Similarly, the magnitude of work-related absenteeism due to WPV varied significantly by HW’s working departments. For example, workplace absenteeism due to WPV was significantly associated with HWs who worked in the surgery department (AOR = 4.11, 95% CI 1.42–11.90; *p* = 0.009), emergency department (AOR = 4.44, 95% CI 1.56–12.61; *p* = 0.005) and gynecology & obstetrics departments (AOR = 5.08, 95% CI 1.47–17.50; *p* = 0.010) compared to HWs who worked at other departments. In addition, the HWs who worked in rotating shifts were significantly 2.61 times more likely to be absent from work due to WPV (AOR = 2.61, 95% CI 1.43–4.75; *p* = 0.002) compared to those who did not work in rotating shifts.

## Discussion

This study provides insight into the impact of WPV on workplace injuries and absenteeism among Bangladeshi HWs. Our results show that workplace injuries and absenteeism due to WPV are prevalent among HWs. In particular, males and young HWs working in emergency, surgery, gynecology & obstetrics departments in public hospitals are at high risk.

The present study found that the magnitude of injuries due to WPV varied significantly by HW’s working departments. For example, the injury incidence due to WPV was substantially higher among HWs who worked in the emergency, surgery, and gynecology & obstetrics departments compared to HWs who worked in the other departments. It’s well documented that the working department in health facilities plays a pivotal role in workplace injuries due to WPV [4–6]. A number of studies highlighted that the following departments are the most vulnerable for serious violence incidents (such as physical violence, and injuries) than the other departments: emergency [5, 6, 11–13], surgery [14], intensive care unit [15] and gynecology & obstetrics departments [32].

The study revealed that the emergency department was the most vulnerable places where the incidence of injury occurred more frequently. This result is consistent with the findings in the USA [13], Finland [23], and Australia [15], where the emergency department HWs experienced more workplace injuries compared to the HWs from other departments. The findings are also in line with a more recent Bangladeshi study that showed the percentage of physical violence at work against doctors was higher for those who worked in the emergency department [7]. The possible reason behind this study's findings could be that the emergency department is the doorway to the inpatient departments, which is why it is the first department to deal with critical patients, followed by the inpatient departments [33]. These patients often die either in an emergency room or inpatient ward despite the best efforts of the HWs. In most cases, patients’ relatives or visitors typically blame HWs for all defects, which in turn exposes them to aggression, and these lead to violent severe incidents such as injury in the workplace. There may also be a lack of health literacy among patients and their relatives. This is because they are unfamiliar with the process of sorting patients’ clinical judgements based on the severity of their health conditions. This process includes difficult decisions by HWs regarding who to provide care for immediately, who can wait, and who cannot be saved [34]. However, patients expect to be seen immediately when they arrive at an emergency department. This leads to a lack of communication is one of the key factors for violent incidents. It is always a challenge in the emergency department to manage heterogeneous patients of various ages, mentalities, and disease severity [12, 35]. Therefore, policymakers need to develop adequate patient protocols for the emergency department, where many people shout out for healthcare and do more to ensure that HWs can work without violent incidents to curb violence. The cause of violent incidents in hospital departments can be investigated in future research, especially workplace injuries in the emergency department.

This study also found that workplace injury was prevalent in the surgery department. This result is consistent with the findings in Australia [14] and India [32], where workplace injury in the surgical department was more prevalent than other forms of violence. The possible reason could be patients with dementia, the emergence of delirium after surgery, or delirium related to drug or alcohol abuse. There is an ongoing threat in the surgical department because HWs must interact with potentially harmful patients or visitors daily. It may result in violent incidents in the surgery department. While available research focuses on the other departments such as the emergency unit [5, 12, 14, 15]. Therefore, it needs to be more research on workplace injuries among HWs due to violent incidents addressing the problem in in-hospital surgical settings. It's unfortunate that workplace injuries due to WPV a tragic realities that extend beyond emergency and surgery departments to sensitive intensive care units. A study conducted in Australia reported that violent incidents were rising in intensive care units [15]. This finding is in line with our results. It could be that increased pressure to admit to the intensive care unit from the emergency department earlier could be a factor contributing to the perceived increase in violent incidents.

According to our study data, we observed a higher rate of absenteeism occurring in the emergency department due to WPV than in other departments. This result is consistent with the findings in the USA [13], China [19], Sweden [36], and Finland [23], where HWs who worked in the emergency department were injured due to WPV taking sick leave or quitting their job. This level of absenteeism is alarming in the emergency department, where severe and emergency cases are treated. Researchers urge that a lack of comprehensive policies for dealing with patients and tackling violent incidents in the emergency department is causing fear, and dissatisfaction among HWs [12, 35], making them absent more often and taking more prolonged periods off-duty due to sickness [5, 19], which has a long-lasting severe negative impact on the health system [18].

A health system’s overall performance is primarily determined by the quality, numbers, composition, and distribution of HWs, which comprise doctors, nurses, midwives and many other categories. It’s pertinent to mention here that Bangladesh still needs more HWs to achieve the recommended global median of 48.6 HWs per 10,000 population by 2030 [37]. A recent WHO report has mentioned that Bangladesh currently presents an estimated 33.17 density of recognised and 15.83 unrecognised HWs and the numbers are unequally distributed across the country’s health system [38]. With this gap, managing a considerable number of patients in the emergency department is strenuous in this growing populated country [39]. Therefore, the lack of human resources for health challenges and gaps needs to be addressed as soon as possible to reduce workplace injuries in hospitals, especially in the emergency department.

The result of this study showed that work-related absenteeism is more prevalent in the gynecology and obstetrics departments. A possible reason behind the findings of this study could be women may lack confidence when defending against violent incidents and fear working in the workplace [40]. In Bangladesh, women are more likely than men to specialise in specific fields due to gender norms, such as gynecology & obstetrics [41]. For these gender norms, the female HWs portion is high in this department, and therefore, women's chances of experiencing WPV are high [24]. Female victims have a high chance of being absent from work due to fear, insecurities and self-image in their workplace. This may lead to an increase in gender imbalance in country’s health system. Therefore, the Bangladesh government must encourage more females to engage in medical careers by raising investment in health resources and minimising public distrust between HWs and the general people. Policymakers should also establish health facilities as safe workplaces for future female health workforces to tackle the country’s shortage of HWs by introducing staff training in skills, cultural diversity, interpersonal communication, and conflict management. In line with existing international findings [14, 15], our study found that the surgery and intensive care departments experienced more workplace absenteeism. It is common for these departments to deal with a high number of critical patients, causing them to feel dissatisfied and stressed out. Hence, the HWs take a break from work and may turn to quit their jobs. In the context of Bangladesh, an in-depth study is required to examine the possible reason for occurring workplace injuries and absenteeism due to WPV in hospital departments.

Notably, the ratio of adequate HWs and patients is going under a very incompatible condition in Bangladesh [28, 38]. For instance, the country’s estimated density of 9.9 doctors, nurses and midwives per 10,000 population in 2019 [42] is far below than the minimum threshold of 44.5 HWs per 10,000 population for the attainment of SDGs outlined in the WHO Global Strategy on Human Resources for Health: Workforce 2030 [43]. In this situation, workplace injuries and absenteeism at work due to WPV will not only increase the crisis but may also cripple the quality health care services in Bangladesh [7, 27]. It is undeniable that workplace injuries and absenteeism due to WPV among HWs partly reflect the disadvantages of Bangladesh’s medical and health systems [7, 27, 28].

The policy implications of our findings are the development of effective national policies to prevent workplace injuries and absenteeism due to WPV among HWs. Our study documented significant injury incidents in the working departments, such as emergency, surgery, intensive care and gynecology and obstetrics departments in Bangladeshi hospitals. The high incidence of injuries in various hospital departments exhibits the country’s lack of ability of a health system to resolve the issue, leading to absenteeism among HWs. These highlight the need for an urgent call for policies and measures for zero tolerance of WPV against Bangladeshi HWs. Therefore, policymakers must approve a law that makes it clear that the law protects the legitimate rights of HWs and their safety must not be infringed upon. For example, we might consider a national ordinance similar to the Nepali government's historic law to protect health workforces named the “Safety and Security of Health Workers and Health Institutions Ordinance, 2022” [21]. In the alternative, we may ratify legislation identical to a Chinese law approved in 2019 that protects HWs from WPV perpetrating serious violence that disturbs medical work [44]. In addition to legislation, much still needs to be done before Bangladeshi HWs can live without WPV. The distrust between patients and HWs has social, cultural, and economic roots and is unlikely to disappear soon. Therefore, the authors stress the importance of breaking down growing communication barriers between patients and HWs by building mutual respect, raising public awareness and representation of stakeholders in the health sector [45]. Furthermore, workplace absenteeism due to injuries as result of WPV among HWs must be stopped immediately, while the country lags behind in the ratio between HWs and population, thereby hindering proper and timely healthcare services [27–29, 42]. If this trend persists, Bangladesh’s health system cannot afford the HWs' absenteeism as they are essential to meet UHC and SDGs target 3 by 2030 [46]. Therefore, the Bangladesh government should establish healthcare institution as a safe workplace for HWs to ensure safe and accessible health care to all and pay attention to strategic investments in the country’s health workforces. Simultaneously, in line with global strategies, policymakers should introduce a specific policy at the national level following international policy recommendations for workplace safety [47]. Finally, the authors emphasise exploring an effective and sustainable solutions for a long-term workplace violence mitigation.

This study has some strengths. First, it used a large, nationally representative dataset from eight administrative division suggesting the findings have external validity [27]. Second, it is the first study to identify the magnitude of injuries and work absenteeism due to WPV by working departments among Bangladeshi HWs. This study also has some limitations. The study did not consider the verbal bulling and harassment as a WPV and their consequences were not justified. As this is cross-sectional study, the findings are correctional only. Finally, there was a possible recall bias in self-reported workplace injuries and absenteeism.

## Conclusions

The study has outlined the prevalence of workplace injuries and absenteeism due to WPV among Bangladesh HWs. This study observed that the magnitude of workplace injury and absenteeism varies among hospital departments. The emergency, surgery, and gynecology & obstetrics departments are more susceptible to workplace injuries and absenteeism due to WPV. Such a high prevalence of workplace injuries and absenteeism negatively impacts the health workforces and the health system. The Bangladesh government must establish health facilities as safe workplaces for HWs where violence against them is not tolerated and approve a law to protect their workplace safety and dignity. Simultaneously, this is essential to pay attention at the national level to increase the availability and accessibility of HWs to ensure access to quality health care for all to meet the 2030 agenda of SDGs target 3. Finally, there is a requirement to examine in depth the reasons and solutions for workplace injuries and absenteeism among HWs to create safe and sustainable workplaces.

### Supplementary Information

**Additional file 1.** Workplace injury and absenteeism due to WPV survey questions used in the current analysis.

## Data Availability

The data used and/or analysed during the current study are available upon reasonable request from the corresponding author.

## Abbreviations

WPV: Workplace violence
HWs: Health workers
WHO: World Health Organization
UHC: Universal Health Coverage
SDGs: Sustainable Development Goals
ILO: International Labor Organization
