# Supplementary material for: Effect of workplace violence on health workers injuries and workplace absenteeism in Bangladesh
Source: Glob Health Res Policy. 2023 Aug 22;8:33. doi: 10.1186/s41256-023-00316-z (PMC10463430; doi:10.1186/s41256-023-00316-z)
Supplement: Supplementary file 1 — Additional file 1. Workplace injury and absenteeism due to WPV survey questions used in the current analysis. [file 41256_2023_316_MOESM1_ESM.docx]

**Supplementary file 1: Workplace injury and absenteeism due to WPV survey questions used in the current analysis**

| **Outcomes** | **Core Question** | **Responses** |
| --- | --- | --- |
| **Workplace injury due to WPV*** | | |
|  | During the past 12 months, were you injured due to WPV? | Percentage of HWs who were injured due to WPV during the 12 months before the survey. |
| **Workplace absenteeism due to WPV**** | | |
|  | During the past 12 months, did you take off from work due to WPV? | Percentage of HWs who took off from work due to WPV during the 12 months before the survey. |
| **Covariates** | | |
|  | Are you? | 1. Male 2. Female |
|  | What is your age in years? |  |
|  | Marital status | 1. Never married 2. Married (including, widowed, divorced, separated) |
|  | Is your hospital? | 1. Public 2. Private |
|  | Working experience in hospital in years |  |
|  | What is your working department? | 1. General medicine 2. General surgery 3. Emergency 4. Intensive care 5. Management 6. Pediatrics and gynae & obs. 7. Others, please specify |
|  | Shift work | 1. No 2. Yes |

**Note: (*):** Workplace injury was defined when a health worker was slight bodily injured, soft tissue contusion, nose bleeding, head trauma, facial blood stasis, waist injury, fracture, concussion, second-/third-degree burn etc.

**(**):** Workplace absenteeism was defined as when a health worker took to take off from/sickness leave due to WPV for any duration.
